# Hospital readmission following transjugular intrahepatic portosystemic shunt: a 14-year single-center experience

**DOI:** 10.1093/gastro/goz062

**Published:** 2019-11-28

**Authors:** Catherine F Vozzo, Tavankit Singh, Jennifer Bullen, Shashank Sarvepalli, Arthur McCullough, Baljendra Kapoor

**Affiliations:** 1 Department of Internal Medicine, Cleveland Clinic, Cleveland, OH, USA; 2 Department of Gastroenterology and Hepatology, Cleveland Clinic, Cleveland, OH, USA; 3 Department of Quantitative Health Sciences, Cleveland Clinic, Cleveland, OH, USA; 4 Department of Inflammation and Immunity, Cleveland Clinic, Cleveland, OH, USA; 5 Department of Radiology, Cleveland Clinic, Cleveland, OH, USA

**Keywords:** transjugular intrahepatic portosystemic shunt, portal hypertension, liver cirrhosis, hospital readmission

## Abstract

**Background:**

Placement of a transjugular intrahepatic portosystemic shunt (TIPS) is a relatively common procedure used to treat complications of portal hypertension. However, only limited data exist regarding the hospital-readmission rate after TIPS placement and no studies have addressed the causes of hospital readmission. We therefore sought to identify the 30-day hospital-readmission rate after TIPS placement at our institution and to determine potential causes and predictors of readmission.

**Methods:**

We reviewed our electronic medical-records system at our institution between 2004 and 2017 to identify patients who had undergone primary TIPS placement with polytetrafluoroethylene-covered stents and to determine the 30-day readmission rate among these patients. A series of univariable logistic-regression models were fit to assess potential predictors of 30-day readmission.

**Results:**

A total of 566 patients were included in the analysis. The 30-day readmission rate after TIPS placement was 36%. The most common causes for readmission were confusion (48%), infection (15%), bleeding (11%), and fluid overload (7%). A higher Model for End-Stage Liver Disease (MELD) score corresponded with a higher rate of readmission (odds ratio associated with each 1-unit increase in MELD score: 1.06; 95% confidence interval: 1.02–1.09; *P *=* *0.001). Other potential predictors, including indication for TIPS placement, were not significantly associated with a higher readmission rate.

**Conclusions:**

The 30-day readmission rate after TIPS placement with covered stents is high, with nearly half of these readmissions due to hepatic encephalopathy—a known complication of TIPS placement. Novel interventions to help reduce the TIPS readmission rate should be prioritized in future research.

## Introduction

Portal hypertension, a known complication of cirrhosis, can lead to variceal bleeding and the development of refractory ascites. In such cases, a transjugular intrahepatic portosystemic shunt (TIPS) can be placed to reduce the pressure in the portal venous system. This procedure can thus be used to manage variceal hemorrhage that cannot be controlled endoscopically, preventing recurrent variceal hemorrhage after multiple endoscopic treatments, as well as treating portal hypertensive gastropathy, refractory ascites, hepatic hydrothorax, and Budd-Chiari syndrome [1, 2].

The first TIPS was placed in 1988 [3]; since that time, the design of the shunt has undergone significant evolution. The Wallstent—the first endovascular stent to be approved by the Food and Drug Administration (FDA)—was limited by poor shunt patency [4, 5]. Viatorr self-expandable polytetrafluoroethylene (PTFE) stents were approved by the FDA in 2004 and have dramatically increased patency rates [4, 6–9]. Additionally, there are only a few absolute contraindications to TIPS placement, making this procedure a viable option for a large number of patients with cirrhosis [10].

Complications related to TIPS placement are relatively common and may be procedural, secondary to shunting, or unique to the shunt itself (e.g. hepatic decompensation, infection, TIPS stenosis) [2, 11–13]. Given such complications, it is not surprising that TIPS would be associated with a high rate of readmissions. One previous study of TIPS placements demonstrated a 30-day readmission rate of 31.3% [14], which is slightly higher than the readmission rate of 26% among all patients with cirrhosis [15]. Because TIPS placement should theoretically control the complications for which patients with cirrhosis are being readmitted, it is surprising that the rate of readmission after TIPS procedures was just as high as, if not higher than, the readmission rate among cirrhotic patients in general. However, this previous study of TIPS placement was limited by a very small sample size of just 83 patients.

In this current study, we used the electronic medical-record system at our institution to determine the 30-day readmission rate in a large population of all patients who had undergone TIPS placement with PTFE-covered stents. We also sought to examine the causes for readmission and attempted to determine whether preventative measures for readmission implemented at our institution would decrease readmission rates.

## Methods

### Study patients

**Figure 1.**
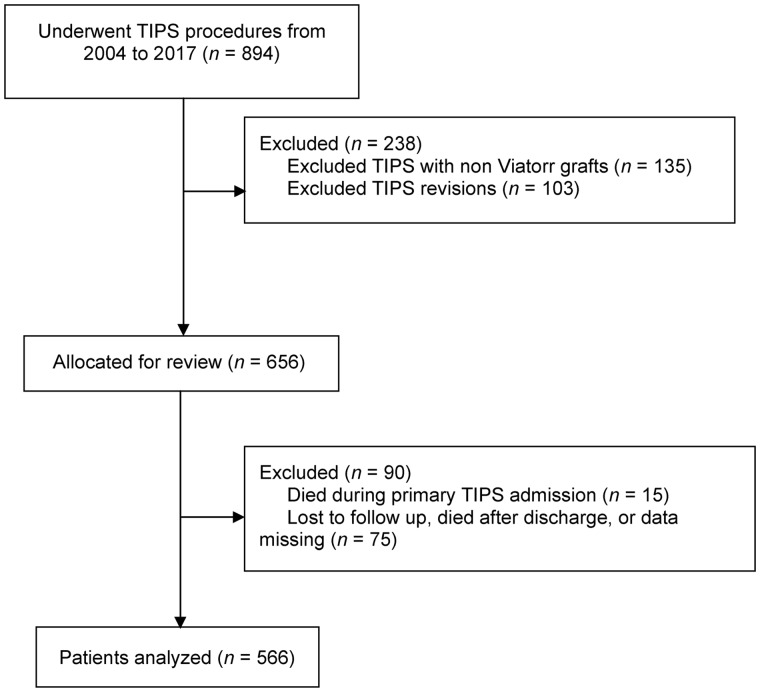
Flow diagram of patient-selection process. TIPS, transjugular intrahepatic portosystemic shunt.

In this retrospective longitudinal observational study, we assessed the medical records of all patients who had undergone TIPS placement at Cleveland Clinic from 2004 through 2017 (PTFE-covered stents were first used at this facility in 2004). Patient data were stored and maintained in a TIPS registry in the REDCap database. Patients were eligible for inclusion if they were aged >18 years, had undergone primary TIPS placement with a PTFE-covered stent for any indication, and had follow-up ≥30 days after the TIPS procedure. Patients who were lost to follow-up or died during admission for primary TIPS placement were excluded from the study. TIPS revisions were also excluded from the analysis, as we were primarily focused on the general risk and reasons for readmission after primary TIPS shunt placement and TIPS revisions may confound the data. See Figure 1 for the complete flow diagram of the patient-selection process. The study design was approved by the local IRB committee.

### Data collection

Information about patient characteristics included age, sex, self-declared race, etiology of liver disease or pre-hepatic portal hypertension, indication for TIPS placement (variceal bleeding, refractory ascites, hepatic hydrothorax, a combination of these indications, or other indication), and the Model for End-Stage Liver Disease (MELD) score. The records were also assessed to determine whether the patient had undergone follow-up liver vascular ultrasonography 4–5 days after TIPS placement, whether a follow-up appointment with a hepatologist had been scheduled for within 2 weeks after hospital discharge, and whether the patient had attended this follow-up appointment. Portosystemic gradient (PSG) values before and after TIPS placement were assessed as follows:

PSG=portal pressure – systemic pressure

Ideal measurement for portal pressure: portal>wedge

Ideal measurement for systemic pressure: right atrium>inferior vena cava>hepatic vein

i.e. *if portal and right atrium are available, PSG = portal* *–* *right atrium*

Information about additional exposures, predictors, and potential confounders was also collected from the medical records; these factors included MELD, liver transplant or hepatic encephalopathy prior to TIPS placement, rationale for TIPS, etiology of liver disease, and whether TIPS placement was emergent or non-emergent. We determined the presence or absence of hepatic encephalopathy before TIPS placement by assessing whether hepatic encephalopathy was listed as a diagnosis, was mentioned in the daily-progress notes, or the patient was receiving therapy for hepatic encephalopathy.

Measures to prevent readmission were reviewed and included whether a hepatology follow-up appointment was scheduled prior to discharge, whether lactulose was prescribed or already a home medication at the time of discharge, and whether a liver vascular ultrasound was completed within the appropriate window (4–5 days after the TIPS placement). The provision of prophylactic lactulose prescription on discharge was dependent on the discharging provider’s discretion, as there is no unified protocol at our institution.

### Outcomes

The primary outcome was readmission to our healthcare system or to a different medical center (with outside electronic medical record available) at 30 days after discharge from the hospitalization during which the TIPS had been placed. The secondary outcome was cause for readmission, which was defined as the chief complaint (or complaints) mentioned at presentation to the emergency department or explained to the provider for direct admission and later validated by the discharge diagnosis. Two reviewers (C.F.V. and T.S.) retrospectively reviewed the medical records to identify both the rate of readmission (primary outcome) and the cause for readmission (secondary outcome).

### Statistical analysis

A series of univariable logistic-regression models were fit to separately assess a set of potential predictors of 30-day readmission status (history of hepatic encephalopathy, percent reduction in PSG, the rationale for TIPS, follow-up vascular ultrasonography in 4–5 days, lactulose prescription given at discharge, on treatment with lactulose or rifaximin prior to TIPS placement, or whether TIPS was emergent or non-emergent). In each model, a Wald test was used to assess the null hypothesis of no association between the predictor and readmission risk. A significance level of 0.05 was applied for each test. Point and interval estimates of the associated odds ratios were also reported. All analyses were performed in R version 3.5.2.

## Results

### Characteristics and demographics

A total of 641 patients underwent TIPS placement with PTFE-covered grafts during the study period. The readmission status at 30 days was unknown for 75 patients in this sample (because of patient death after index hospitalization or because patients were lost to follow-up), so only 566 patients were included in the analysis.

As shown in Table 1, the mean age was 56 years at the time of TIPS and 55% of this cohort was male. The two most common etiologies of liver disease in the study population were non-alcoholic steatohepatitis (27%) and alcoholic liver disease (24%). A TIPS was most commonly placed to treat ascites (37%) or variceal bleeding (33%). The size of the TIPS stent was most commonly 10 mm in diameter 90.8% (514/566), followed by 12 mm 0.07% (44/566), and 8 mm 0.01% (8/566).

**Table 1.** Summary of baseline characteristics among 566 patients undergoing TIPS placement with PTFE-covered stents*

Characteristic	Patients NOT readmitted within 30 days (*n* = 364)	Patients readmitted within 30 days (*n* = 202)
Mean age at time of TIPS placement, years	56.04 ± 11.19	55.93 ± 10.86
Male	200 (54.9)	114 (56.4)
Liver-disease etiology		
Non-alcoholic steatohepatitis	94 (25.8)	59 (29.2)
Alcoholic liver disease	93 (25.5)	42 (20.8)
Cryptogenic causes	27 (7.4)	13 (6.4)
Hepatitis B	5 (1.4)	2 (1.0)
Hepatitis C	44 (12.1)	29 (14.4)
Hepatitis B + hepatitis C	1 (0.3)	0 (0.0)
Hepatitis B + alcoholic liver disease	1 (0.3)	0 (0.0)
Hepatitis C + alcoholic liver disease	34 (9.3)	19 (9.4)
Hepatitis B + hepatitis C + alcoholic liver disease	3 (0.8)	1 (0.5)
Miscellaneous**	54 (14.8)	31 (15.3)
Missing/unknown	8 (2.2)	6 (3.0)
Indication for TIPS		
Ascites	137 (37.6)	74 (36.6)
Hydrothorax	18 (4.9)	9 (4.5)
Variceal bleed	124 (34.1)	65 (32.2)
Ascites + hydrothorax	30 (8.2)	15 (7.4)
Ascites + variceal bleed	30 (8.2)	22 (10.9)
Variceal bleed + hydrothorax	1 (0.3)	2 (1.0)
Ascites + variceal bleed + hydrothorax	3 (0.8)	2 (1.0)
Other	21 (5.8)	13 (6.4)
Mean MELD score at time of TIPS placement	11.83 ± 4.64	13.33 ± 5.73
History of hepatic encephalopathy before TIPS placement	114 (31.6)	78 (38.6)
Mean percent reduction in PSG	63.87 ± 17.20	61.88 ± 17.80
Follow-up vascular ultrasonography performed within 4–5 days	124 (34.2)	76 (38.2)
Follow-up hepatology appointment scheduled within 2 weeks	143 (39.6)	77 (38.5)
Received lactulose prescription at discharge	200 (55.6)	113 (55.9)
Treated with lactulose before TIPS placement	104 (28.9)	68 (34.0)
Treated with rifaximin before TIPS placement	51 (14.1)	37 (18.5)
Emergency TIPS placement	54 (15.2)	28 (14.1)

Data are presented as mean ± standard deviation or *n* (%).

TIPS, transjugular intrahepatic portosystemic shunt; PTFE, polytetrafluoroethylene; MELD, Model for End-Stage Liver Disease; PSG, portosystemic gradient.

* Some information was unknown in some patients, including MELD score (*n *=* *3), history of hepatic encephalopathy (*n *=* *3), PSG reduction (*n *=* *3), ultrasonography follow-up status (*n *=* *4), hepatology follow-up status (*n *=* *5), lactulose prescription status (*n *=* *4), pre-TIPS lactulose status (*n *=* *6), pre-TIPS rifaximin status (*n *=* *5), and emergency TIPS placement status (*n *=* *11).

** Miscellaneous etiologies of liver disease included: autoimmune hepatitis, primary biliary cirrhosis, hemochromatosis, Wilson’s disease, alpha-1 antitrypsin, granulomatous disease, drug-induced liver disease, and venous outflow obstruction.

The mean MELD score at the time of TIPS placement was 12 and the mean reduction in PSG was 63%. Of note, hepatic encephalopathy was present prior to TIPS placement in 34% of the patients who were treated with lactulose, rifaximin, or both. Less than 40% of the patients received a follow-up liver vascular ultrasound or had a follow-up office visit scheduled at the time of discharge.

### Clinical outcomes

**Figure 2.**
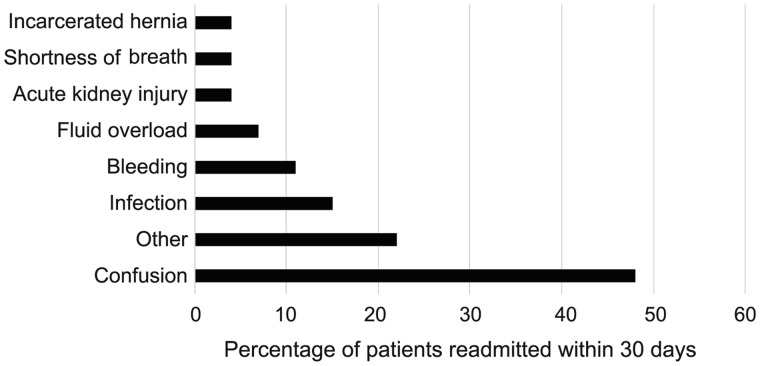
Causes for 202 cases of readmission at 30 days after transjugular intrahepatic portosystemic shunt. Note that patients could have more than one cause for readmission. The category ‘Other’ encompasses several causes for readmission, including incarcerated abdominal hernia, abdominal pain, nausea/vomiting, falls, and pancreatitis.

The overall 30-day readmission rate was 36% (202/566). The most common cause for readmission at 30 days was confusion related to hepatic encephalopathy (48%). Other common causes for readmission included infection (15%), bleeding (11%), and fluid overload (7%) (Figure 2).

Patients with higher MELD scores at the time of TIPS placement had a higher risk of 30-day readmission (odds ratio [OR] associated with each 1-unit increase in MELD score: 1.06, 95% confidence interval [CI]: 1.02–1.09; *P *=* *0.001). The readmission rate was 33% (67/204) among patients with a MELD score <9, 33% (99/297) among patients with a MELD score of 10–19, and 58% (36/62) among patients with a MELD score of 20–30. Other than the MELD score at the time of the TIPS, there were no other significant predictors of 30-day readmission (Table 2). Patients with a history of hepatic encephalopathy before TIPS placement had a trend for higher rates of 30-day readmission (OR = 1.36, 95% CI: 0.95–1.95; *P *=* *0.092). The readmission rate was 41% (78/192) among patients with a history of encephalopathy and 33% (124/371) among patients without a history of encephalopathy. Among patients without a history of encephalopathy, there was no significant difference in readmission rates in patients who were treated with lactulose prophylactically vs those not treated with lactulose prophylactically (29% [41/139] vs 36% [83/232]; OR = 0.75; 95% CI: 0.47–1.18; *P *=* *0.215). The degree of PSG reduction also did not affect readmission rates (OR = 0.99, 95% CI: 0.98–1.00; *P *=* *0.196).

**Table 2.** Potential predictors of 30-day readmission among 566 patients undergoing TIPS placement with PTFE-covered stents

Variable	Odds ratio	95% CI	*P*-value
History of hepatic encephalopathy before TIPS placement (yes vs no)	1.36	0.95–1.95	0.092
MELD score at time of TIPS placement (increase of 1)	1.06	1.02–1.09	0.001
Reduction in PSG (increase of 1%)	0.99	0.98–1.00	0.196
Ascites as an indication for TIPS placement (yes vs no)	1.04	0.74–1.47	0.819
Variceal bleed as an indication for TIPS placement (yes vs no)	1.07	0.76–1.51	0.706
Both ascites and variceal bleed as indications for TIPS placement (yes vs no)	1.35	0.77–2.35	0.288
Follow-up vascular ultrasonography performed within 4–5 days (yes vs no)	1.19	0.83–1.70	0.340
Follow-up hepatology appointment scheduled within 2 weeks (yes vs no)	0.95	0.67–1.36	0.796
Lactulose prescription given at discharge (yes vs no)	1.02	0.72–1.44	0.930
Treated with lactulose before TIPS placement (yes vs no)	1.27	0.87–1.84	0.209
Treated with rifaximin before TIPS placement (yes vs no)	1.38	0.86–2.19	0.174
Emergency TIPS placement (yes vs no)	0.92	0.55–1.19	0.727

TIPS, transjugular intrahepatic portosystemic shunt; PTFE, polytetrafluoroethylene; MELD, Model for End-Stage Liver Disease; PSG, portosystemic gradient; CI, confidence interval.

**Figure 3.**
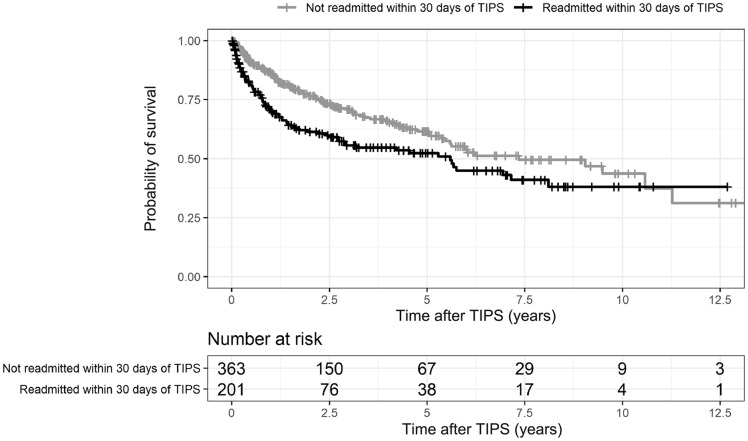
Kaplan–Meier survival curve for patients who were readmitted within 30 days after transjugular intrahepatic portosystemic shunt (TIPS) and for patients who were not readmitted within 30 days.

Survival status at 90 days after TIPS placement was known for 512 patients (54 patients did not have sufficient follow-up). Patients who were readmitted within 30 days of TIPS placement were approximately three times more likely to die within 90 days of TIPS placement than those who were not readmitted within 30 days (OR = 3.6, 95% CI: 1.8–7.2; *P *<* *0.001). The 90-day mortality rate was 14% (25/183) among patients readmitted within 30 days and 4% (14/329) among patients not readmitted within 30 days (Figure 3).

## Discussion

In recent years, the costs of healthcare have come under increased scrutiny. The cost of TIPS placement, for instance, has been reported to be increasing across the USA [16]. This increased focus on costs was reinforced with the passage of the Affordable Care Act. This act, among other initiatives, established the Hospital Readmissions Reduction Program with the aim of improving quality of care and reducing overall costs. Currently, hospitals are penalized for excessive readmissions within 30 days of discharge for certain conditions and these conditions may soon expand to include complications of radiology-guided procedures such as TIPS placements [14, 15]. Thus, radiology staff must be well informed regarding the readmission rates and causes for readmission at their healthcare institutions.

In this study, we found that patients undergoing TIPS placement with PTFE-covered stents had a 30-day hospital-readmission rate of 36%. This readmission rate is surprisingly high when compared with the 30-day readmission rate for patients with cirrhosis in general (26%) [15]. The readmission rate we observed is also higher than the rate reported in a previous study of readmissions after TIPS placement (31%) [14]. One possible explanation for the higher readmission rate we observed after TIPS placement relates to the type of patient population our healthcare institution treats; as a quaternary referral center, our institution often receives high-risk transfers from outside facilities that are unable to perform successful TIPS placement.

Nearly half (48%) of the 30-day readmissions in our study were for confusion related to hepatic encephalopathy, a well-known complication of TIPS placement. Previous studies have shown that, in patients with cirrhosis who have not undergone TIPS placement, the rate of readmission for hepatic encephalopathy is much lower (22% and 35%) [17, 18].

Unsurprisingly, we found that a higher MELD score carried a higher risk of readmission after TIPS placement. The mean MELD score for patients not readmitted was 12 vs a mean MELD score of 13 for patients who were readmitted (*P *<* *0.05). However, the percent reduction in PSG was not associated with hospital-readmission rates; this is surprising, given that lower PSG leads to higher rates of hepatic encephalopathy [19, 20].

We also examined protective factors that may prevent readmission after TIPS placement. These factors included giving patients without a history of hepatic encephalopathy prophylactic lactulose, with instructions to begin taking the medication upon development of any signs or symptoms of hepatic encephalopathy. Other protective factors involved scheduling a follow-up hepatology appointment within 2 weeks after patients were discharged and performing liver vascular ultrasonography within the next 5–7 days. These factors did not appear to affect the rate of readmission in our study population. However, all providers do not routinely provide a prophylactic lactulose prescription on discharge and we were unable to determine whether all lactulose prescriptions were filled or whether the medication was taken when early signs and symptoms of encephalopathy were noted. This highlights the importance of providing patients and caregivers with counseling on the early signs and symptoms of encephalopathy and offering a trial of lactulose treatment after discharge.

This study was limited by potential sources of error common among retrospective chart reviews, including recall bias, misclassification bias, and confounding. However, this study did include only those patients who were treated with covered stents, which should have eliminated any potential bias from including older stents that were more prone to occlusion and complications. Additionally, results regarding preventative factors must be interpreted with caution, as there was a fair amount of heterogeneity among providers caring for patients who were admitted after TIPS placement for observation. Not all providers followed the same protocol of prescribing prophylactic lactulose, arranging 2-week hepatology follow-up visits, or ensuring that liver vascular ultrasonography was scheduled. Finally, our institution is a quaternary referral center and our results may not be generalizable to the medical community at large.

In conclusion, this study is the first to review readmission rates after TIPS placements with covered stents and is the first to identify the various causes for readmission in a large patient population. Future studies should review these causes for readmission (especially the most common, hepatic encephalopathy) and assess various initiatives that might decrease readmission rates after TIPS placements. Future studies might also evaluate the possible link between the presence of spontaneous portosystemic shunts and increased readmissions or complications after TIPS procedures [21]. Finally, to reduce the risk of readmission after TIPS placements, healthcare providers should be sure to provide counseling to patients and caregivers about the early signs and symptoms of hepatic encephalopathy, thus allowing early identification of the condition and timely treatment initiation.
